# 1759. Maintaining Latin America Polio-Free: A Multidimensional Analysis of Polio Outbreak Risk in Argentina in the Period 2020-2022

**DOI:** 10.1093/ofid/ofad500.1590

**Published:** 2023-11-27

**Authors:** Veronica Lucconi Grisolia, Rocio Nahir Barrios, Janisse Requena Olavarría, Juan León Cañete, María Jimena Aranda, Nathalia Katz, Raquel Gabriela Elbert, Silvina Neyro, Maria Eugenia Urruti, María Victoria López, Carolina Selent, Octavia Bertachini, Ana Martina De Prada, Solana Rapaport, Adriana Verónica Hernández, Andrés Martín Pereira, Florencia Bruggesser

**Affiliations:** Ministry of Health of Argentina, Ciudad Autónoma de Buenos Aires, Ciudad Autonoma de Buenos Aires, Argentina; Ministry of Health of Argentina, Ciudad Autónoma de Buenos Aires, Ciudad Autonoma de Buenos Aires, Argentina; Ministry of Health of Argentina, Ciudad Autónoma de Buenos Aires, Ciudad Autonoma de Buenos Aires, Argentina; Ministry of Health of Argentina, Ciudad Autónoma de Buenos Aires, Ciudad Autonoma de Buenos Aires, Argentina; Ministry of Health of Argentina, Ciudad Autónoma de Buenos Aires, Ciudad Autonoma de Buenos Aires, Argentina; Ministry of Health of Argentina, Ciudad Autónoma de Buenos Aires, Ciudad Autonoma de Buenos Aires, Argentina; Ministry of Health of Argentina, Ciudad Autónoma de Buenos Aires, Ciudad Autonoma de Buenos Aires, Argentina; Ministry of Health of Argentina, Ciudad Autónoma de Buenos Aires, Ciudad Autonoma de Buenos Aires, Argentina; Ministry of Health of Argentina, Ciudad Autónoma de Buenos Aires, Ciudad Autonoma de Buenos Aires, Argentina; Ministry of Health of Argentina, Ciudad Autónoma de Buenos Aires, Ciudad Autonoma de Buenos Aires, Argentina; Ministry of Health of Argentina, Ciudad Autónoma de Buenos Aires, Ciudad Autonoma de Buenos Aires, Argentina; Ministry of Health of Argentina, Ciudad Autónoma de Buenos Aires, Ciudad Autonoma de Buenos Aires, Argentina; Ministry of Health of Argentina, Ciudad Autónoma de Buenos Aires, Ciudad Autonoma de Buenos Aires, Argentina; Ministry of Health of Argentina, Ciudad Autónoma de Buenos Aires, Ciudad Autonoma de Buenos Aires, Argentina; Ministry of Health of Argentina, Ciudad Autónoma de Buenos Aires, Ciudad Autonoma de Buenos Aires, Argentina; Ministry of Health of Argentina, Ciudad Autónoma de Buenos Aires, Ciudad Autonoma de Buenos Aires, Argentina; Ministry of Health of Argentina, Ciudad Autónoma de Buenos Aires, Ciudad Autonoma de Buenos Aires, Argentina

## Abstract

**Background:**

Argentina has eliminated the circulation of poliovirus (PV) in 1984, and from 2020 onward only inactivated vaccines (IPV) are included in its national immunization schedule (NIS) which consist of three doses and a booster. The persistence of PV in some countries and the regional use of attenuated vaccines allow for the risk of reintroduction of these viruses. Such risk can be estimated for each population based on the susceptibility to infection, the capabilities to identify an event and prevent its spread. Our main objective was to analyze the PV outbreak risk in Argentina since the exclusive use of IPV.

**Methods:**

Descriptive study. We used the PV outbreak risk analysis model provided by the Regional Certification Commission for the Polio Endgame in the Region of the Americas (RCC). The data of the period 2020-2022 available in the records of the Ministry of Health of the Nation were synthesized. It includes the following dimensions: polio vaccination coverage, epidemiological surveillance, social determinants of health and the occurrence of other vaccine-preventable diseases.

**Results:**

We analyzed the PV outbreak risk in the 512 departments of the country in 2020, 2021 and 2022. For each year, respectively, 83% (n: 425), 86.3% (n: 442) and 94.1% (n: 482) of the departments had high and very high risk. In these departments, the dimension of polio vaccination coverage contributed predominantly to the risk, with an average of 52.6% (SD: 8.9); 57.9% (SD: 7.9); 58.1% (SD: 8.6) in each series. In regards to the coverage in the last 5 years, with the third dose of IPV, 73.4%, 61.3% and 60.8% of the departments reached the average expected. In respect of the coverage with the second dose of IPV, only 26.6%, 16.7% and 29.5% reached as expected for each year. During the vaccination campaign 26.3% of the departments achieved the coverage expected.

**Figure:**
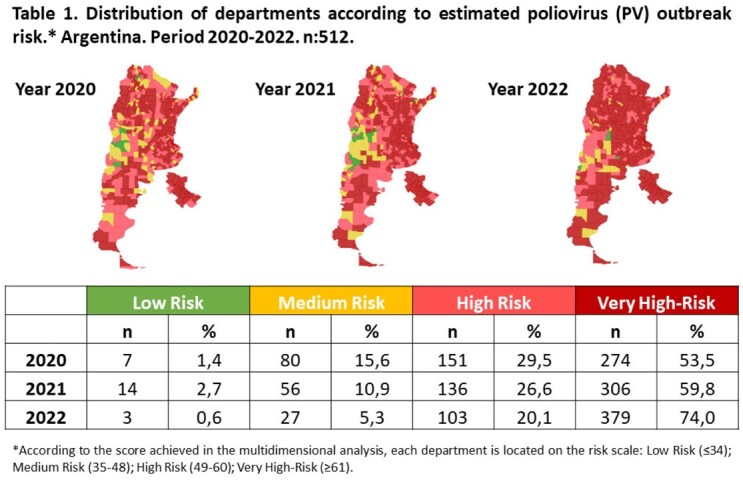

**Figure:**
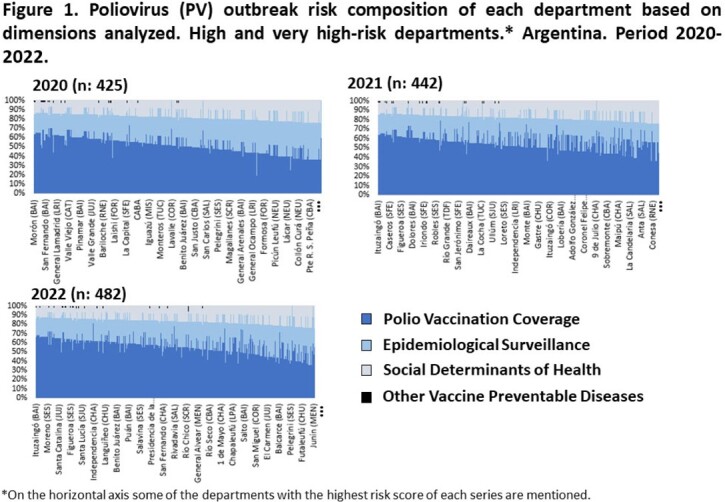

**Figure:**
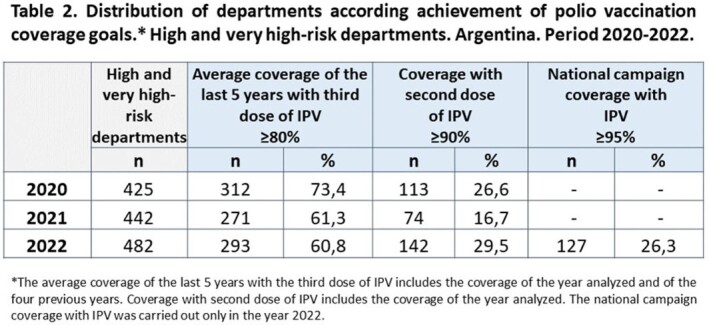

**Conclusion:**

Though the exclusive use of IPV in the NIS has represented a big step for Argentina, the multidimensional risk analysis shows a high PV outbreak risk, similar to that described for other Latin American countries. This is an essential tool in the making of national polio elimination programs. We must continue working in order to meet polio vaccination coverage goals and keep the population disease-free.

**Disclosures:**

**All Authors**: No reported disclosures

